# Amelioration of CCl_4_-induced oxidative stress and hepatotoxicity by *Ganoderma lucidum* in Long Evans rats

**DOI:** 10.1038/s41598-023-35228-y

**Published:** 2023-06-19

**Authors:** Fatima Tuj Johra, Sukria Hossain, Preeti Jain, Anika Tabassum Bristy, Tushar Emran, Rezwana Ahmed, Shazid Md Sharker, Asim Kumar Bepari, Hasan Mahmud Reza

**Affiliations:** Department of Pharmaceutical Sciences, North South University, Dhaka, 1229 Bangladesh

**Keywords:** Biochemistry, Biological techniques, Drug discovery, Medical research, Drug development, Experimental models of disease, Preclinical research

## Abstract

Liver disease is a serious health problem affecting people worldwide at an alarming rate. The present study aimed to investigate the protective effects of *Ganoderma lucidum* against CCl_4_-induced liver toxicity in rats. The experimental Long Evans rats were divided into five groups, of which four groups were treated with carbon tetrachloride (CCl_4_). Among the CCl_4_ treated groups, one of the groups was treated with silymarin and two of them with ethanolic extract of *G. lucidum* at 100 and 200 mg/Kg body weight. The oxidative stress parameters and endogenous antioxidant enzyme concentrations were assessed by biochemical tests. Liver enzymes ALT, AST, and ALP were determined spectrophotometrically. Histopathological examinations were carried out to assess hepatic tissue damage and fibrosis. Reverse transcription PCR (RT-PCR) was performed to determine the expression of IL-1β, IL-6, IL-10, TNF-α, and TGF-β genes. Gas Chromatography-Mass Spectroscopy (GC–MS) analysis revealed that *G. lucidum* is rich in several phytochemicals including 6-Octadecanoic acid (55.81%), l-( +)-Ascorbic acid 2,6-dihexadecanoate (18.72%), Cis-11-Eicosenamide (5.76%), and Octadecanoic acid (5.26%). Treatment with the *G. lucidum* extract reduced the elevated ALT, AST, ALP levels, and cellular oxidative stress markers and increased the endogenous antioxidant levels. Histopathology observations revealed that the inflammation, infiltration of immune cells, and aberration of collagen fibers in the hepatocytes were altered by the *G. lucidum* treatment. The increased expression of inflammatory cytokines TNF-α, TGF-β, IL-1 β, and IL-6 were markedly suppressed by *G. lucidum* extract treatment. *G. lucidum* also prevented the suppression of protective IL-10 expression by CCl_4_. This study strongly suggests that *G. lucidum* extract possesses significant hepatoprotective activity as evidenced by reduced oxidative stress and inflammation mediated by suppression in inflammatory cytokine expression and increased protective IL-10 cytokine expression.

## Introduction

Oxidation is an essential step of cellular metabolism that leads to the generation of free radicals and reactive oxygen species (ROS) both physiologically and pathologically. These highly reactive radicals may accelerate the oxidation of biomolecules in the cells and tissues of living organisms leading to damage and death. Uncontrolled generation of free radicals is involved in the progression of many diseases such as liver cirrhosis, atherosclerosis, arthritis, especially rheumatoid arthritis, cancer, and many degenerative processes, including aging^1^. Endogenous oxidative enzymes like superoxide dismutase (SOD), glutathione peroxidase, and catalase (CAT) capture the free radicals and convert them into less reactive ones, thereby protecting against oxidative damage^2^. Along with endogenous compounds, some exogenous chemicals such as α-tocopherol, ascorbic acid, carotenoids, and polyphenol compounds also play essential roles in scavenging the free radicals, breaking down the free radicals chain reaction, and neutralizing the reactive oxygen species^3,4^. Oxidative damage by pathological or age-related causes cannot be overcome easily when there is an imbalance in the endogenous protective compounds in the body. In this situation, an intake of antioxidants containing supplements and foods becomes essential^5^.

The liver is the primary organ for metabolizing nutrients, drugs, and xenobiotics mainly by a family of enzymes called cytochrome P-450^6^. Carbon tetrachloride (CCl_4_) is a well-known exogenous hepatotoxic agent. Free radical generation during the hepatic metabolism of CCl_4_ by cytochrome P-450 leads to lipid peroxidation, covalent bonding of radicals with the structural proteins, nuclear DNA and mitochondrial DNA, and aberration of DNA. CCl_4_ activates the resident macrophages of the liver known as Kupffer cells that produce and secrete chemoattractants and neutrophil activators which causes the recruitment of neutrophils into that site. Neutrophils release reactive oxygen species and cause inflammatory changes in the hepatocytes leading to hepatotoxicity^7^. Endogenous and exogenous antioxidants can capture the free radicals and prevent hepatocellular damage^8^.

Besides nutritionally beneficial primary compounds such as protein, vitamins and minerals, mushrooms also contain many secondary constituents, including phenolic compounds such as alkaloids, terpenes, and steroids. Mushrooms help in lowering blood pressure, blood glucose and cholesterol levels, and are found useful in the treatment of diabetes, atherosclerosis, hypercholesterolemia, heart diseases, osteoporosis and peptic ulcer^9–11^. Hepatoprotective, anti-inflammatory, antibacterial, antiviral, immunomodulatory, and anticancer properties of various types of mushrooms have been reported previously^11–13^. Approximately 10,000 different species of mushrooms have been identified^14–17^. People in many Asian countries take the fruit body of *G. lucidum* to treat coronary diseases, arteriosclerosis, hepatitis, arthritis, nephritis, bronchitis, asthma, hypertension, cancer, and gastric ulcer^18^. The varied ethnopharmacological uses of *G. lucidum* have inspired researchers to analyze the chemical constituents in its extract.

Gas chromatography-mass spectrometry (GC–MS) is one of the highly reliable techniques to identify the bioactive compounds such as phenolics, terpenoids, long chain and branched chain hydrocarbons, alcohols, alkaloids, organic acids and esters^19^. However, for better evaluation of chemical profile of compounds and investigation of triterpenoid patterns present in *G. lucidum*, multiple methods have been adopted such as RP-HPLC, HPLC–UV-ESI–MS, NMR and FT-IR^19–21^. Triterpenoids and polysaccharides are the two important pharmacologically active principles found in the fruiting bodies of *G. lucidum*. Polysaccharides obtained from *G. lucidum* are known to exhibit a wide range of pharmacological effects such as antioxidant, immunomodulatory, neuroprotective, antidiabetic, anti-inflammatory and anticancer activities^22,23^. Pure β-glucans, heterofucans, heteromannans and their complexes with peptides have been isolated from *G. lucidum* fruiting bodies and the pure glucose polymer and the polymer built of β-D-glucose and α-D-galactose have shown antioxidant activities^24^. Protection against reactive oxygen species (ROS) and liver damage has been demonstrated by crude polysaccharides extracted from *G. lucidum*^22,23^. The effects of total triterpenoid extracts from mushrooms were studied in experimental liver injury models induced by hepatotoxic agents such as ethanol, carbon tetrachloride and D-galactosamine in mice^13^. Several studies have reported the effectiveness of triterpenoids against hepatotoxicity, tumor, hypertension, hypercholesterolemia, and platelet aggregation^18^. Mushrooms are also rich in phenolics and flavonoids which contribute towards the antioxidant effects together with other health benefits^12,13^. Investigation of antioxidant activity through multiple in vitro models have demonstrated significant free radical scavenging activities of *G. lucidum* extracts^10^ which could help in improving oxidative stress-associated diseases such as diabetes, degenerative disorders, stroke and liver diseases. Against this background, the present study aims to evaluate the potential of *G. lucidum* extract in ameliorating carbon tetrachloride-induced hepatotoxicity and associated oxidative stress in Long Evans rats.

## Results

### Gas chromatography-mass spectroscopy (GC–MS) analysis

**Figure 1:**
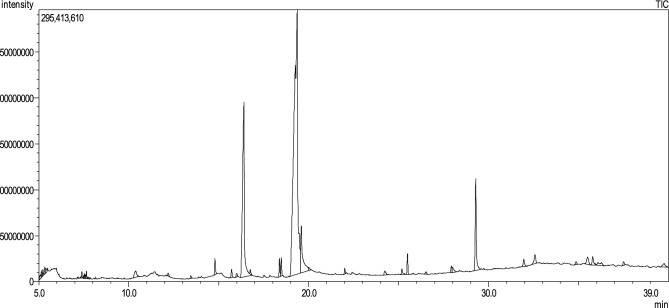
GC chromatogram of the ethanolic extract of *G. lucidum* powder.

We analyzed the ethanolic extract of *G. lucidum* powder using a triple-quad GC–MS/MS system as shown in Fig. 1. Thirty-seven phytochemicals were identified in the chromatogram which are tabulated along with retention time (Ret Time), molecular formula, molecular weight and area (%) (Table 1). The most abundant compound was 6-Octadecanoic acid with a peak area of 55.81%. Other notable compounds were l-( +)-Ascorbic acid 2,6-dihexadecanoate (18.72%), Cis-11-Eicosenamide (5.76%), and Octadecanoic acid (5.26%).

**Table 1:** Compounds identified by GC–MS/MS analysis of the ethanolic extract of *G. lucidum* powder.

S/N	Ret. time	Area (%)	Chemical name	Nature	Molecular weight	Molecular formula
1.	5.085	0.27	Benzene, propyl-	Alkylbenzene	120.19	C_9_H_12_
2.	5.153	0.21	Benzene, 1-ethyl-3-methyl-	Alkylbenzene	120.19	C_9_H_12_
3.	5.334	0.36	Hexanoic acid	Fatty acid	116.16	C_6_H_12_O_2_
4.	5.405	0.26	1-Pentene, 5-chloro-4-(chloromethyl)-	Alkene	153.05	C_6_H_10_Cl_2_
5.	7.485	0.1	Nonanoic acid	Fatty acid	158.24	C_9_H_18_O_2_
6.	7.532	0.15	2-Octene-1-ol, 3,7-dimethyl-	Alcohol	156.26	C_10_H_20_O
7.	7.607	0.09	Acetic acid, 2,2’-(3,6-dimethoxy-1,4-dioxane-2,5-diylidene)bis-,dimethyl ester	Dimethyl ester	288.25100	C_12_H_16_O_8_
8.	7.654	0.24	Cyclohexanol, 5-methyl-2(1-methylethyl)-,(a.alpha.,2.beta.,5alpha)-(. + / − .)-	Cyclohexanols	156.26	C_10_H_20_O
9.	7.789	0.11	D-Arabino-Hexose, 2-deoxy-,diethyl mercaptal	Mercaptals	270.4	C_10_H_22_O_4_S_2_
10.	10.381	0.79	1,3-Propandiol, 2-(hydroxymethyl)-2-nitro-	Polyols	151.11	C_4_H_9_NO_5_
11.	12.198	0.15	Ethyl .alpha-d-glucopyranoside	O-glycosyl compounds	208.21	C_8_H_16_O_6_
12.	13.542	0.12	Tetradecanoic acid	Fatty acid	228.37	C_14_H_28_O_2_
13.	14.793	0.76	Pentadecanoic acid	Fatty acid	242.40	C_15_H_30_O_2_
14.	15.722	0.46	Triacontanoic acid	Fatty acid	452.8	C_30_H_60_O_2_
15.	16.003	0.23	Palmitoleic acid	Fatty acids, Monounsaturated	254.41	C_16_H_30_O_2_
16.	16.406	18.72	l-( +)-Ascorbic acid 2,6-dihexadecanoate	Fatty acid esters	652.9	C_38_H_68_O_8_
17.	16.759	0.2	Hexadecanoic acid, ethyl ester	Fatty acid ethyl ester	284.5	C_18_H_36_O_2_
18.	18.379	0.81	Methyl 10-trans, 12-cis-octadecadienoate	Fatty acid methyl ester	294.5	C_19_H_34_O_2_
19.	18.483	0.81	9-Octadecanoic acid, methyl ester, (E)-	fatty acid methyl ester	296.48	C_19_H_36_O_2_
20.	19.363	55.81	6-Octadecanoic acid	Fatty acids	282.46	C_18_H_34_O_2_
21.	19.589	5.26	Octadecanoic acid	Fatty acid	284.47	C_18_H_36_O_2_
22.	20.03	0.17	n-Pentadecanol	Long-chain fatty alcohol	228.41	C_15_H_32_O
23.	24.243	0.39	Carbamic acid, 2-(dimethylamino)ethyl ester	Quaternary ammonium compounds	132.16	C_5_H_12_N_2_O_2_
24.	25.183	0.38	Hexadecanoic acid, 2-hydroxy-1-(hydroxymethyl)ethyl ester	1-monoacylglycerols	330.50	C_19_H_38_O_4_
25.	25.494	0.99	Bis(2-ethylhexyl) phthalate	Phthalate ester	390.55	C_24_H_38_O_4_
26.	26.526	0.16	Lanosterol	Tetracyclic triterpenoid	426.71	C_30_H_50_O
27.	27.928	0.39	6,9-Octadecadienoic acid, methyl ester	Fatty acid methyl ester	294.50	C_19_H_34_O_2_
28.	28	0.49	2,3-Dihydroxypropyl elaidate	Monoacylglycerol	356.50	C_21_H_40_O_4_
29.	29.286	5.76	Cis-11-Eicosenamide	Primary fatty amide	309.50	C_20_H_39_NO
30.	31.962	0.55	Ergosta-5,7,9(11),22-tetraen3-ol,(3.beta.,22E)-	Phytosterol	394.63	C_28_H_42_O
31.	32.581	0.85	20.xi.-Lanosta-7,9(11)-dien-21-oic acid, 16.alpha.-hydroxy-24-methylene-3-oxo-	Lanostane triterpene	482.69	C_31_H_46_O_4_
32.	34.861	0.24	Ergosta-5,7,9(11)-tetraen-3-ol,(3.beta.,22E)-	Phytosterol	394.63	C_28_H_42_O
33.	35.504	0.81	Ergosterol	Phytosterol	396.64	C_28_H_44_O
34.	35.788	0.68	Ergosta-5,7-dien-3-ol,(3.beta.)-	Phytosterol	398.66	C_28_H_46_O
35.	36.087	0.76	Rhodopin	Carotenoid	554.88	C_40_H_58_O
36.	37.505	0.29	gamma.-Sitosterol	Phytosterols	414.70	C_29_H_50_O
37.	39.754	0.5	Retinol	Retinoids	286.45	C_20_H_30_O

### Effect of *G. lucidum* extract on the body weight

**Figure 2:**
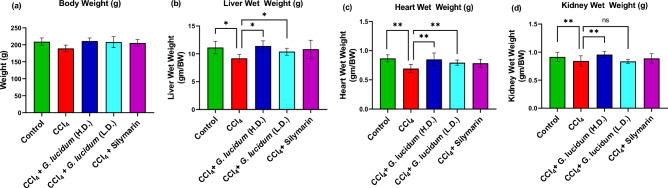
Effect of *G. lucidum* extract supplementation on the body weight (**a**) and the organ wet weight (**b**–**d**) in different groups. Statistical analysis was carried out by one‐way ANOVA followed by Newman–Keul’s post hoc test. Statistical significance was considered at *p ≤ 0.05 and **p ≤ 0.01, *ns* not significant. *HD* high dose (200 mg/kg), *LD* low dose (100 mg/kg).

All the rats were weighed routinely throughout the experimental period. As shown in Fig. 2a, no statistically significant difference in body weights was noticed among different experimental groups. This shows that carbon tetrachloride, silymarin, or the sample extract did not adversely affect the body weight of the experimental animals.

### Effect of *G. lucidum* extract on organ wet weight

Statistically significant differences in the liver, heart, and kidney weights of rats have been observed between different groups of experimental animals (*p ≤ *0.05). The disease group (CCl_4_ treated rats) showed the lowest organ weights among all the groups. As shown in Fig. 2b, the control group, the disease-treatment (high dose) group, and the standard silymarin group showed similar liver weight. The carbon tetrachloride group had a significant reduction in liver weight. The rats of the disease group (CCl_4_ treated group) also showed a significant decrease in heart (Fig. 2c) weight compared to the control and disease treatment groups. As shown in Fig. 2d, a significant decrease in kidney weight was obseved in disease group compared to the control and high dose-disease treatment groups. However, low dose extract treatment did not show any significant differnce in kidney weight as compared to the disease group.

### Effect of *G. lucidum* extract on ALP, ALT, and AST activities

**Figure 3:**
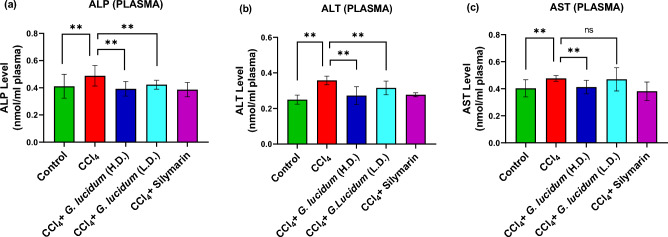
Effect of *G. lucidum* extract supplementation on ALP (a), ALT (b), and AST (c) enzyme activities in plasma of CCl_4_ administered rats. Statistical analysis was carried out by one‐way ANOVA followed by Newman–Keul’s post hoc test. Statistical significance was considered at *p ≤ 0.05 and **p ≤ 0.01, *ns* not significant. *HD* high dose (200 mg/kg), *LD* low dose (100 mg/kg).

A significantly high plasma level of ALP and ALT was observed in the disease group (CCl_4_ treated group) compared to the other groups (*p* ≤ 0.01) (Fig. 3). Lower levels of ALP and ALT were observed with both low and high dose treatment groups compared to the disease group (Fig. 3a,b). The high dose treatment group exhibited a lower AST level (*p* ≤ 0.01) whereas low dose treatment did not, compared to the disease group (Fig. 3c).

### Effect of *G. lucidum* extract on oxidative stress parameters

**Figure 4:**
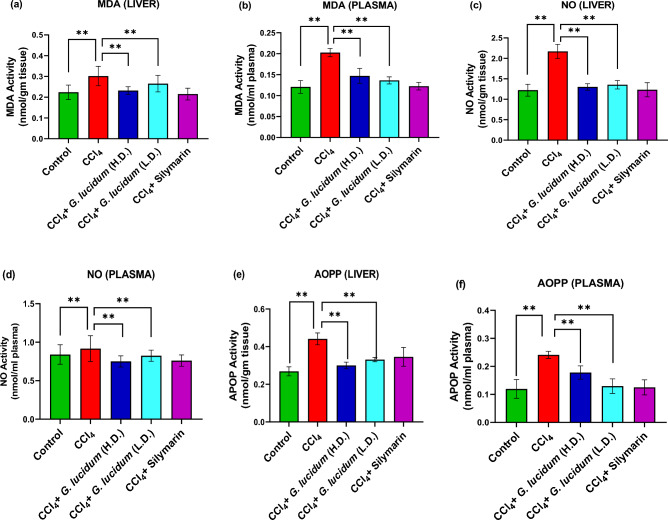
Effect *G. lucidum* powder extract on MDA (**a**, **b**), NO (**c**, **d**), and AOPP (**e**, **f**) activities in liver and plasma of CCl_4_ administered rats. Statistical significance was considered at *p ≤ 0.05, **p ≤ 0.01. *HD* high dose (200 mg/kg), *LD* low dose (100 mg/kg).

Significant elevation in malondialdehyde (MDA) level was observed in the disease group compared to the control (*p* ≤ 0.01) (Fig. 4a,b). In contrast, the liver and plasma samples from the high dose treatment group showed a significant decrease in MDA level compared to the disease group (*p* ≤ 0.01). MDA level was also significantly reduced in the standard group (silymarin treatment) compared to the disease group, and the reduction was more than in both extract treatment groups. A higher concentration of MDA was observed in plasma than in liver homogenate.

The level of NO in the liver homogenate (Fig. 4c) of the disease group was much higher than the control, standard, and extract treatment groups. At the same time, it was almost identical among the control, standard, low dose, and high dose treatment groups. As shown in Fig. 4d, the highest level of NO was observed in plasma (Fig. 4d) of the disease group whereas, high dose treatment and standard treatment groups showed much lower levels of plasma NO (*p* ≤ 0.01).

The Fig. 4e shows that ethanolic extract of *G. lucidum* (200 mg/kg) significantly (*p* ≤ 0.01) diminished the level of AOPP in the liver as compared to the disease group. A similar change was observed in the standard group treated with silymarin and the low-dose treatment group. However, high dose treatment showed better effect compared to the low dose treatment. On the other hand, low dose treatment was found to be more effective in reducing plasma AOPP levels (Fig. 4f) compared to the high dose treatment.

### Effect of *G. lucidum* extract on antioxidant enzymes activities

**Figure 5:**
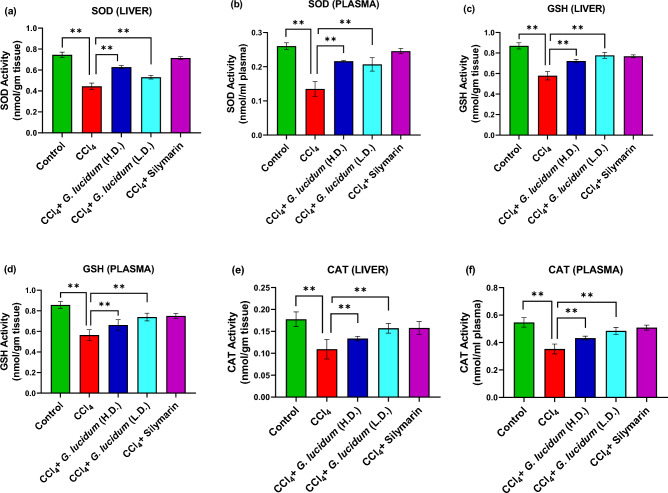
Effect of *G. lucidum* extract supplementation on SOD (**a**, **b**), GSH (**c**, **d**) and CAT (**e**, **f**) activities in liver and plasma of CCl_4_ administered rats. Statistical significance was considered at *p ≤ 0.05, **p ≤ 0.01. *HD* high dose (200 mg/kg), *LD* low dose (100 mg/kg).

Extract and standard treatment groups showed increased SOD, GSH, and CAT levels in both liver homogenate and plasma samples compared to the disease group (Fig. 5).The increase in SOD level was higher in the standard group (silymarin treatment) than in the extract treatment groups (Fig. 5a,b). As shown in (Fig. 5a), the higher dose of *G. lucidum* extract increased the SOD level more than the low dose treatment in liver homogenate.

GSH level was found to be higher in the standard and the extract treatment groups, both in liver homogenate and plasma (Fig. 5c,d) as compared to the disease group. The low dose and standard treatment groups showed similar levels of GSH (Fig. 5c,d). A higher CAT level was observed in the standard and low dose treatment group than in the high dose treatment group in both liver homogenate and plasma (Fig. 5e,f).

### Histopathological observation

**Figure 6:**
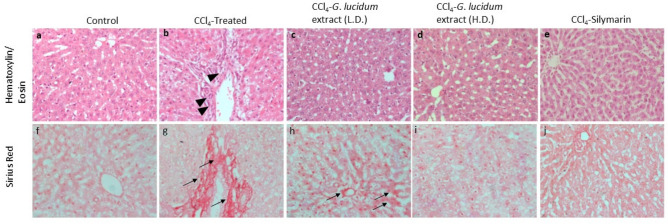
Histopathological observations showing the effects of CCl_4_-induced hepatotoxicity and the hepatoprotective effect of *G. lucidum* extract (Low dose: 100 mg/kg and High dose: 200 mg/kg). The upper panel (**a**–**e**) shows hematoxylin/eosin staining and the lower panel (**f**–**j**) shows Sirius Red staining. Arrowheads indicate infiltrating cells (**b**) and arrows indicate collagen deposition (**g**, **h**).

The hematoxylin/eosin staining of the liver tissue revealed regular internal cellular morphological structure (Fig. 6a). No abnormalities, infiltration of immune cells (e.g., mononuclear cells), and tissue necrosis were observed in the internal structure of the control group (Fig. 6a). Infiltration and necrosis were observed in the disease group (CCl_4_ treated group) (Fig. 6b, arrowheads), which was altered in the low dose (Fig. 6c) and high dose- (Fig. 6d) mushroom extract-treated groups. The standard silymarin-treated rats showed regular tissue architecture (Fig. 6e).

The Sirius red staining of the liver tissues showed normal distribution of collagen fibers in the control group (Fig. 6f). Intense collagen and fatty infiltration distribution were observed in the CCl_4_ treated disease group indicating significant fibrosis (Fig. 6g, arrows). The low dose mushroom-treated group exhibited slightly higher collagen deposition (Fig. 6h, arrows) than the control. However, the collagen distribution was nearly normal with high dose mushroom or silymarin treatment (Fig. 6i,j respectively). Quantification data is provided in Figure S1.

### Gene expression analysis

**Figure 7:**
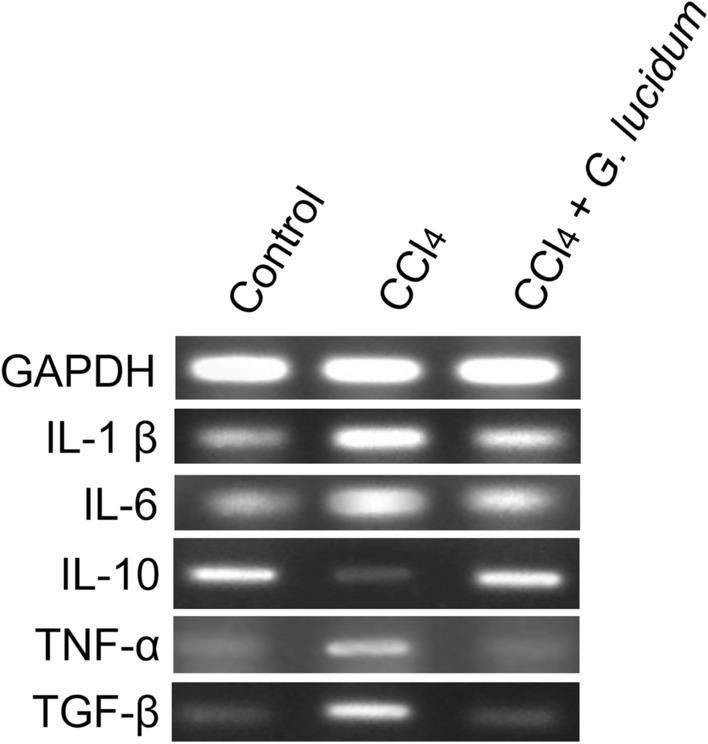
Expression of a housekeeping gene (GAPDH) and cytokine genes (IL-1β, IL-6, Il-10, TNF-α, TGF-β) in liver tissues collected from different groups.

Prominent expression of the housekeeping gene GAPDH was observed in the control, disease, and disease-treatment (High dose) group (Fig. 7, Figure S2). The administration of CCl_4_ or mushroom extract did not affect GAPDH gene expression and acted as an internal control. The expression of IL-1β, an inflammatory cytokine, was observed in carbon tetrachloride treated groups, which was suppressed by the extract treatment. The expression of IL-6 was also observed in the disease group that the extract treatment suppressed. IL-10 is an inflammation protector. Administration of carbon tetrachloride suppressed this hepatoprotective IL-10 gene expression, whereas a higher level of IL-10 gene expression was observed in the extract group. This indicates that supplementation with *G. lucidum* extract can protect against inflammation. Higher expression of inflammatory cytokine TNF-α was observed in the disease group compared to the control and treatment groups. The results support that Ganoderma extract has an ameliorative effect on hepatotoxicity as exhibited by lower expression of TNF-α. CCl_4_-induced hepatotoxicity increased TGF-β expression, which is responsible for liver fibrosis. In this study, the disease group showed TGF-β expression, which was reduced by treatment with the extract (High dose).

## Discussion

While nutritional benefits of *G. lucidum* have been known for centuries, a recent focus is the exploration of medicinal applications of the extract and isolated phytochemicals. Phytochemical analysis using GCMS revealed that *G. lucidum* ethanolic extract contains bioactive compounds (Table 1) with pharmacological effects such as antioxidant, anti-inflammatory, anticancer, antimicrobial, and hepatoprotective effects. l-( +)-Ascorbic acid 2,6-dihexadecanoate (18.72%) which was identified as one of the major constituents is known to be a potent antioxidant with hepatoprotective^25^ effects. Triterpenoids, which were found in the mushroom extract are reported to provide protection against liver injury induced by toxic chemicals such as CCl_4_, thioacetamide and alcohol^13^. Triterpenoids are emerging as a unique group of phytochemicals with multifunctional activities like anti-adipogensis, anti-inflammatory, analgesic, antipyretic, cardiotonic, sedative and hepatoprotective effects^9,26^. Ergosterols present in the mushroom extract as evidenced by GCMS analysis, have been shown to exert anti-tumor, anti-angiogenic^21^ and protective effects against liver fibrosis ^11^. Several studies have demonstrated the hepatoprotective and antioxidant effects of retinoid^27,28^. 9-Octadecenoic acid and its esters have been shown to exhibit antiandrogenic, 5-alpha reductase-inhibiting, cancer-preventive, anti-inflammatory, antioxidant and hypocholesterolemic activity^12,19,29^. Hexadecanoic acid detected in the mushroom extract has also been reported to have antioxidant, hypocholesterolemic, antibacterial and anticancer activities^12,19,29^. Furthermore, 6-Octadecenoic acid, a major constituent (55.81%), detected in the *G. lucidum* has been shown to possess anti-cancer and antimicrobial activities^19^.

In this study, the hepatoprotective effects of *G. lucidum* extract were explored in a rat model of carbon tetrachloride-induced hepatic damage. The effects were compared with the standard silymarin. Administration of carbon tetrachloride significantly reduced the internal organs’ weight, whereas co-administration of extract and CCl_4_, or silymarin and CCl_4_ didn’t show any significant reduction in the organs’ weights (Fig. 2). This is in accordance with previous studies^30,31^.

CCl_4_ administration raised hepatic AOPP, MDA, and NO levels and increased lipid peroxidation leading to antioxidant defense failure and hepatic damage^32^. ROS are often generated at physiological and pathological levels, either due to cellular metabolism or in response to an insult to the body's homeostasis. Excess free radicals cause oxidative stress indicators like MDA, AOPP, and NO to rise^33^. Increased ROS level degrades membrane phospholipids and decreases antioxidant levels in the affected tissues^34^. In this study, CCl_4_-induced hepatic damage raised the level of stress biomarkers which included MDA, AOPP, and NO. In contrast, extracts and standard treatment groups exhibited decreased stress biomarkers. Oxidative stress triggers the mineralocorticoid receptor activation^35^, leading to reduced levels of SOD, GSH, and CAT enzymes. These enzymes are part of the body’s first-line defense against oxidative stress and scavenge the ROS to sustain the physiological condition.

Protective effect of *G. lucidum* extracts has been demonstrated against hepatotoxicants such as ethanol, cyclophosphamide, benzo[a]pyrene and CCl_4_ in experimental animals at varying concentrations^13,36–39^. Lin et al. (1995)^37^ reported 66% hepatoprotection by aqueous extract of *G. lucidum* at 100 mg/kg dose whereas, methanolic extract of *G. lucidum* at 500 mg/ kg dose has been shown to prevent benzo[a]pyrene induced hepatic damage in rats^40^. Ameliorative effect of *G. lucidum* on carbon tetrachloride-induced liver fibrosis in rats has been demonstrated previously at a dose of 600 mg/kg^38^ and 2000 mg/kg crude extract^36^. However, in this study ethanolic extract of locally grown *G. lucidum* showed significant hepatoprotection in rats at 200 mg/kg dose. The findings of this study indicate that the fruiting bodies of *G. lucidum* occurring in Bangladesh, could present promising candidate for the development of effective liver protective agents.

In this study, the disease group exhibited a decrease in the levels of SOD, CAT, and GSH compared to the control, which was significantly (*p* ≤ 0.01) protected with the ethanolic extract of *G. lucidum* (EEGL) supplementation. Sirius-red and Hematoxylin/ Eosin staining revealed that these biochemical alterations led to histopathologic changes (Fig. 6). In the liver tissue of diseased (CCl_4_-treated) rats, we found altered hepatic architecture, fibrosis, and a buildup of darkly stained nuclei. The oxidative state, hepatic enzyme activity, and histological alterations were apparently undisturbed when EEGL or silymarin was given concurrently (Fig. 6). Similar results were reported by previous studies with plant extracts^30,41,42^.

Any pathological damage to the hepatocytes releases the liver marker enzymes into the blood circulation, e.g., Alanine aminotransferase (ALT), Aspartate aminotransferase (AST), Alkaline Phosphate (ALP), etc. The level of ALT, AST, ALP, bilirubin, and low-density lipoprotein (LDH) in the blood rises as the hepatocytes are destroyed. The results obtained from the biochemical analysis in our study are in line with the previous reports^43–45^ that showed an increase in the ALT, AST, ALP, lactate dehydrogenase concentration, and a decrease in the concentration of glutathione peroxidase, SOD, and CAT in CCl_4_-induced hepatotoxicity in male Wistar rats. In the current study, the mushroom extract provided noticeable protection against the elevation of liver marker levels in test animals.

For a better understanding of the efficacy of *G. lucidum* extract, expression analysis of some housekeeping, inflammatory, and liver regeneration associated genes has been carried out. The housekeeping gene GAPDH expression showed no change in any group, which indicates that the Ganoderma extract or CCl_4_ did not affect the expression of the housekeeping gene. Eltahir et al.^46^ reported an increase in the expression of several inflammatory cytokine genes associated with CCl_4_ administration in rats. Interleukin 6 (IL-6), tumor necrosis factor-alpha (TNF-α) mRNA expression, necrosis factor-kappa B (NF-κB) proteins level, TGF-β, COX-2 expression has also been observed in the CCl_4_-induced hepatic injury in rats^46^. In our study, increased expression of inflammatory cytokines TNF-α, TGF-β, IL-1 β, and IL-6 due to CCl_4_ was suppressed by the ethanolic extract of *G. lucidum*. Additionally, the CCl_4_-mediated decrease in expression of the inflammatory protector IL-10 gene was restored by the extract treatment. Our findings demonstrate that *G. lucidum* is effective in preventing the elevated level of liver markers thereby ameliorating oxidative stress in hepatic injury. At a molecular level, it showed a reduction in expression of the studied inflammatory gene and an increase in the hepatoprotective genes.

Our findings demonstrate that *G. lucidum* could be used as a promising hepatoprotective agent and in the treatment of hepatic fibrosis. GCMS analysis suggests that presence of hepatoprotective compounds such as ascorbic acid, triterpenoids, ergosterols and retinol together with numerous other bioactive molecules may contribute towards hepatoprotective effect of mushroom. This study also provides valuable insights into mechanisms that underlie the hepatoprotective effect of *G. lucidum*, supposedly mediated through attenuation of lipid peroxidation, restored activities of antioxidant enzymes, downregulation of inflammatory cytokines genes and upregulation of the protective genes like IL-10. Phytochemical diversity of secondary metabolites indicates potential of *G. lucidum* for the development of newer drugs. Further investigations with isolated pure compounds are desirable to elucidate the precise therapeutic potential of *G. lucidum* for clinical applications.

## Conclusions

In our study, CCl_4_ reliably induced hepatotoxicity and oxidative stress in Long Evans rats. The disease group displayed elevated MDA, AOPP, and NO levels and attenuated SOD, CAT, and GSH levels. Interestingly, supplementation with the *G. lucidum* extract or the standard antioxidant silymarin efficiently protected the experimental rats from oxidative stress-induced pathological changes. These findings were further supported by gene expression analysis and histopathological studies. Thus, it would be interesting to explore the antioxidant and hepatoprotective properties of the isolated phytochemicals from *G. lucidum* in future studies.

## Materials and methods

### Chemicals

Carbon tetrachloride (CCl_4_) was purchased from Qualigens Fine Chemicals, India. Silybin R (Silymarin, reference drug) capsules were obtained from Square Pharmaceuticals Ltd., Bangladesh. Kits used in the determination of serum alanine aminotransferase (ALT), aspartate aminotransferase (AST), and alkaline phosphatase (ALP) were purchased from Human Diagnostics, Germany. GoScriptTM Reverse Transcription System (Promega) was used to perform the reverse transcription process. All other chemicals and solvents were of the analytical grade and obtained from Sigma or Merck.

### Extract preparation

Dried *G. lucidum* powder was obtained from whole *G. lucidum* (Figure S3) as described previously^10^ and extracted with ethanol. Extraction was carried out approximately for seven days, accompanied by occasional shaking and stirring. The mixture was filtered first through a piece of clean, white cotton material and finally through Whatman No-1 filter paper. The extract was evaporated to dryness under reduced pressure by a rotary vacuum evaporator followed by overnight incubation at room temperature to ensure complete evaporation of residual solvents. The dried extract was stored at 4 °C in a refrigerator until used for analysis^39^.

### Gas chromatography-mass spectroscopy (GC–MS) analysis

Phytochemicals of the dried *G. lucidum* powder extract were analyzed using a GC–MS/MS system (GCMS-TQ8040, Shimadzu). Chromatographic separation was carried out in a capillary column (Rxi-Sms, 30 m × 0.25 mm × 0.25 μm) using helium (99.99% pure, flow rate: 1.2 ml/min) as the carrier gas. The column oven temperature was 75 °C and the injection temperature was 250 °C. MS conditions were as follows: ion source temperature 230 °C; interface temperature 250 °C; detector gain 1.13 kV + 0.2 kV; end time 35 min; Acq. mode Q3 Scan; event time 0.5 s; scan speed 2000; m/z range 50–1000, injection volume 0.5 μl (10 μg/ml).

### Experimental animals

Thirty Long Evans rats (average weight 190 ± 10 g), 10–12 weeks old, collected from the Animal House of the Department of Pharmaceutical Sciences, North South University, were used in this study. The experimental inclusion criteria of the test animals were specific parameters such as body weight, fitness, and health. The animals were kept in individual cages under standard conditions (12 h light/dark cycle; 23–26 °C) with free access to standard diet and water. The overall study protocol complied with the standards for animal care and experimentation of Institutional Animal Care and Use Committee (IACUC) of North South University in accordance with the guidelines from the Council for International Organization of Medical Sciences and The International Council for Laboratory Animal Science (CIOMS/ICLAS, 2012) and the Nuffield Council on Bioethics (NCB). The study was reported in accordance with ARRIVE guidelines^47^.

### Study design and treatment

Thirty male Long Evans rats selected for the experiment were divided into five experimental groups, each consisting of six rats. The treatment period was two weeks for all the groups, and all the substances were administered intragastrically. The groups and their specifications have been mentioned in Table 2.

**Table 2:** Study groups and the specifications of each group.

Group name	Group type	Treatment specification
Group I	Negative control	1 mL/Kg body weight of saline (0.9% Sodium chloride solution) and 1 mL/kg olive oil twice a week
Group II	Disease group	CCl_4_ at a dose of 1 mg/Kg body weight dissolved in olive oil (1:3 ratio) twice a week
Group III	Standard group	CCl_4_ at a dose of 1 mg/Kg body weight dissolved in olive oil (1:3 ratio) twice a week + silymarin 50 mg/Kg body weight daily
Group IV	Disease-treatment group (low dose)	CCl_4_ at a dose of 1 mg/Kg body weight dissolved in olive oil (1:3 ratio) twice a week + mushroom extract at a dose of 100 mg/Kg body weight daily
Group V	Disease-treatment group (high dose)	CCl_4_: olive oil (1:3 ratio) at a dose of 1 mL/Kg body weight twice a week + mushroom extract at a dose of 200 mg/Kg body weight daily

**Figure 8:**
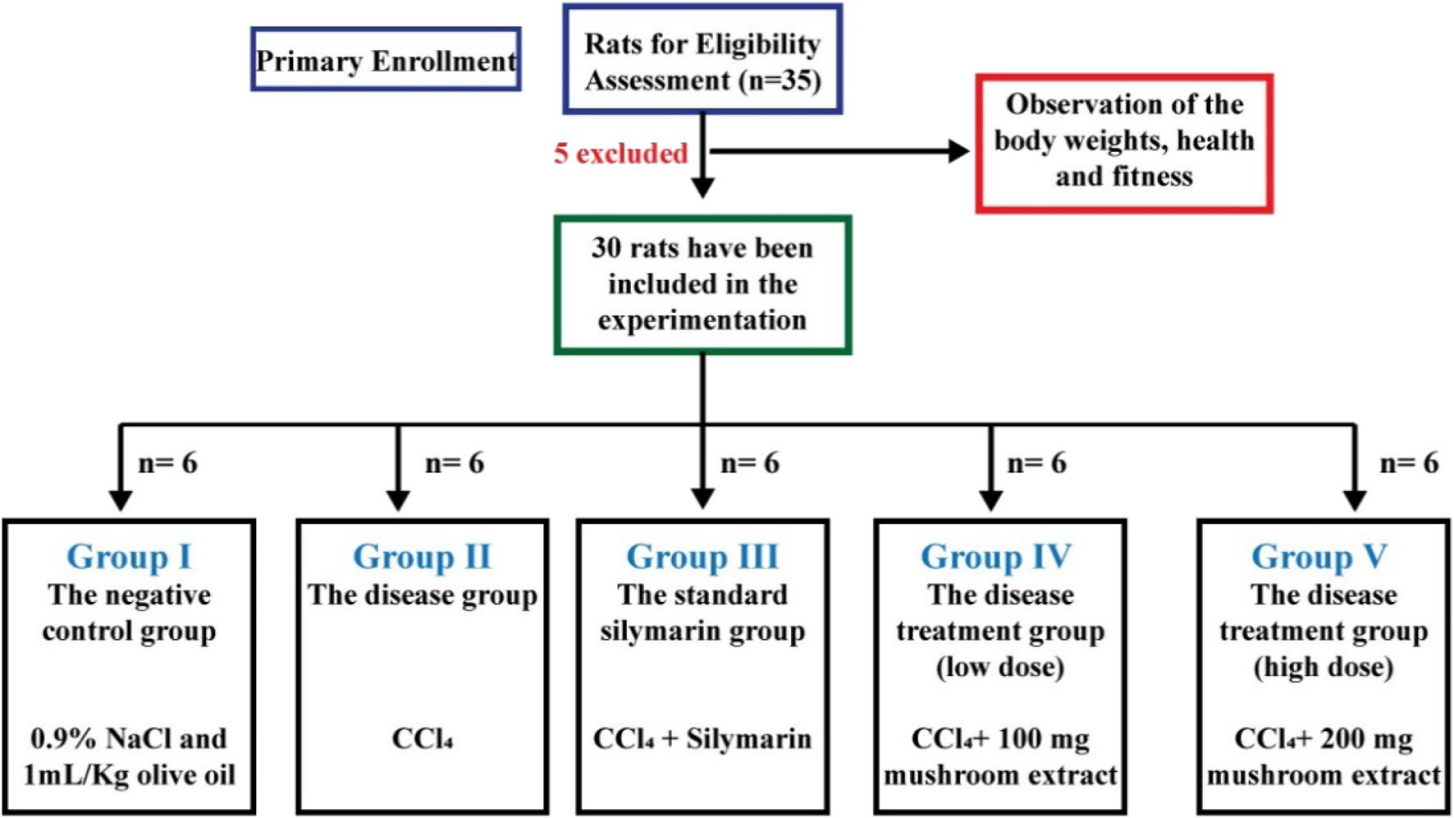
The inclusion and exclusion of the test animals and the experimentation groups.

The inclusion and exclusion of the test animals and the overall experimentation groups, along with the test criteria of each group, have been shown diagrammatically in Fig. 8.

The test agents were administered intragastrically using a feeding needle and a 10 mL syringe to measure the volume accurately. The rats' body weight, food, and water intake were checked daily. After two weeks of treatment, the animals of all groups were sacrificed after anesthetization with intraperitoneal ketamine hydrochloride injection. 3 mL blood samples from each animal were collected in citrate buffer tubes and centrifuged at 2000 rpm for 15 min at 4 °C for serum separation. Plasma was transferred in pre-labeled eppendorf tubes and stored at − 20 °C for further analysis. The liver and other organs such as the heart, spleen, and kidney were harvested. For histopathological analysis, parts of each rat liver were stored in the 10% formalin (pH 7.4). For gene expression analysis in hepatocytes, specimens from the liver were cut into pieces and stored at − 20°C^30^.

### Body weight and organ weight determination

The rats of all groups were weighed regularly, and the average weight of each group was determined to assess the impact of treatments and test chemicals on the body weight. After dissection, the weight of collected organs such as liver, kidney, heart, and spleen was recorded, and variation among each group was analyzed.

### Biochemical parameters

#### Hepatotoxicity assessment

Quantitative determination of the liver function marker enzymes; alanine aminotransferase (ALT), aspartate aminotransferase (AST), and alkaline phosphatase (ALP) in plasma was carried out with a spectrophotometer using commercial diagnostic kits according to the manufacturer’s protocol.

#### Estimation of lipid peroxidation

Lipid peroxidation and oxidative stress in plasma and liver tissue were determined colorimetrically by measuring thiobarbituric acid reactive substances (TBARS). The sample (tissue homogenate/blood plasma) was treated with phosphate buffer solution (PBS), glacial acidic acid (AA), and thiobarbituric acid (TBA 0.67%) and boiled for 30 min. For homogenates, the ratio of PBS, AA, and TBA was 4:5:5, whereas, for plasma, it was 1:2:2. This was followed by measuring the absorbance of the cooled and clear supernatant at 490 nm wavelength^48^. The extent of lipid peroxidation was expressed as MDA equivalents.

#### Estimation of nitric oxide (NO)

This assay is based on the fact that nitric oxide present in the sample solution undergoes diazotization with sulfanilamide followed by coupling with N-(1-naphthyl) ethylenediamine hydrochloride (NED). Briefly, 20 µL tissue homogenate or 50 µL plasma samples were taken in a 96-well plate. This was followed by mixing with 0.33% (w/v) sulphanilamide and 0.1% (w/v) N-(1-naphthyl) ethylenediamine hydrochloride in phosphate buffer solution. Then absorbance was measured at 405 nm wavelength. NO level was determined using a standard curve made by serial dilution of 1 mM sodium nitrite stock solution^49^.

#### Determination of advanced oxidation protein products (AOPP)

The tissue homogenate/plasma sample was taken with phosphate buffer solution (PBS) and mixed with 0.45% (w/v) potassium iodide and glacial acidic acid. The mixture was left for 2 min at room temperature to complete the reaction. Absorbance was recorded at 405 nm wavelength^50^.

#### Reduced glutathione assay (GSH)

The tissue homogenate or plasma sample was mixed with phosphate buffer solution (1:9). This was followed by adding 100 µL 5, 5-dithiobis-2-nitrobenzoic acid (DTNB) and incubating for 1 h at 4 °C. Absorbance was measured at 450 nm wavelength^51^.

#### Catalase assay (CAT)

Tissue homogenate or plasma sample was taken in a 96-well culture plate, and phosphate buffer was added. This was followed by adding the appropriate concentrations of hydrogen peroxide solution to the mixture. Absorbance was recorded at 450 nm wavelength three times at 3 min intervals^52^.

#### Determination of superoxide dismutase (SOD)

The tissue homogenate/plasma sample (10 µL) was added to a 90 µL phosphate buffer solution. This was followed by adding 100 µL adrenaline and immediately measuring absorbance at 490 nm wavelength. A control reaction consisting of all the ingredients except the tissue/plasma was analyzed simultaneously^53^. SOD activity was determined in terms of inhibiting autooxidation of adrenaline to adrenochrome.

### Gene expression profile

#### RNA isolation and purification

Total RNA was isolated from frozen liver tissue samples using Trizol reagent^54^. RNase-free reagents were used throughout the process, and the samples were treated with DNase I to avoid contamination of genomic DNA. Isolated RNA was dissolved in RNAase-free water and stored at − 20 °C. The yield and integrity of isolated RNA were evaluated by measuring absorbance at 260, 280, and 310 nm and agarose gel (1%) electrophoresis.

#### Reverse transcription and PCR analysis

RNA samples were reverse-transcribed to first-strand complementary DNA (cDNA) using GoScriptTM Reverse Transcription System (Promega) according to the manufacturer’s instructions. For semi-quantitative analysis of specific mRNA, 4 µL of cDNA template was mixed with 10 µL of PCR master mix, 4 µL nuclease-free water, and 1 µL of (100 nmol/mL) forward and reverse primers (Table S1) in PCR tubes and the mix was put into the TaKaRa PCR Thermal Cycler (TP650, TaKaRa, Japan). The following conditions were used: initial denaturation at 94 °C for 5 min, 30 cycles of denaturation at 94 °C for 30 s, annealing at the corresponding temperature for 30 s, extension at 72 °C for 30 s, and final extension at 72 °C for 7 min. Finally, the gene expression was analyzed by agarose gel electrophoresis.

### Histopathological studies

Liver tissues excised from different groups of animals were fixed in neutral buffered formalin (10%), dehydrated in graded alcohol, and embedded in paraffin. Microtome Sects. (0.5 µ) were prepared and stained with hematoxylin/eosin and Sirius red stains. This was followed by observation under a light microscope (Olympus DP12, Japan) at 20 × magnification to assess histopathological changes^30^.

### Statistical analysis

All data were presented as the Mean ± Standard Error of Mean (SEM) (n = 6). The results were subjected to analysis of variance (ANOVA) by using Graph pad Prism 8. Values were considered statistically significant at *p* ≤ 0.05.

### Ethical approval

The animal study protocol was approved by the Institutional Animal Care and Use Committee (IACUC) of North South University (IACUC Id: 2020/OR-NSU/IACUC-No.1105) for studies involving animals.

## Supplementary Information

Supplementary Information.

## Data Availability

The datasets used and/or analyzed during the current study are available from the corresponding author on reasonable request.
