# Supplementary material for: Amelioration of CCl4-induced oxidative stress and hepatotoxicity by Ganoderma lucidum in Long Evans rats
Source: Sci Rep. 2023 Jun 19;13:9909. doi: 10.1038/s41598-023-35228-y (PMC10279652; doi:10.1038/s41598-023-35228-y)
Supplement: Supplementary file 1 — Supplementary Information. [file 41598_2023_35228_MOESM1_ESM.pdf]

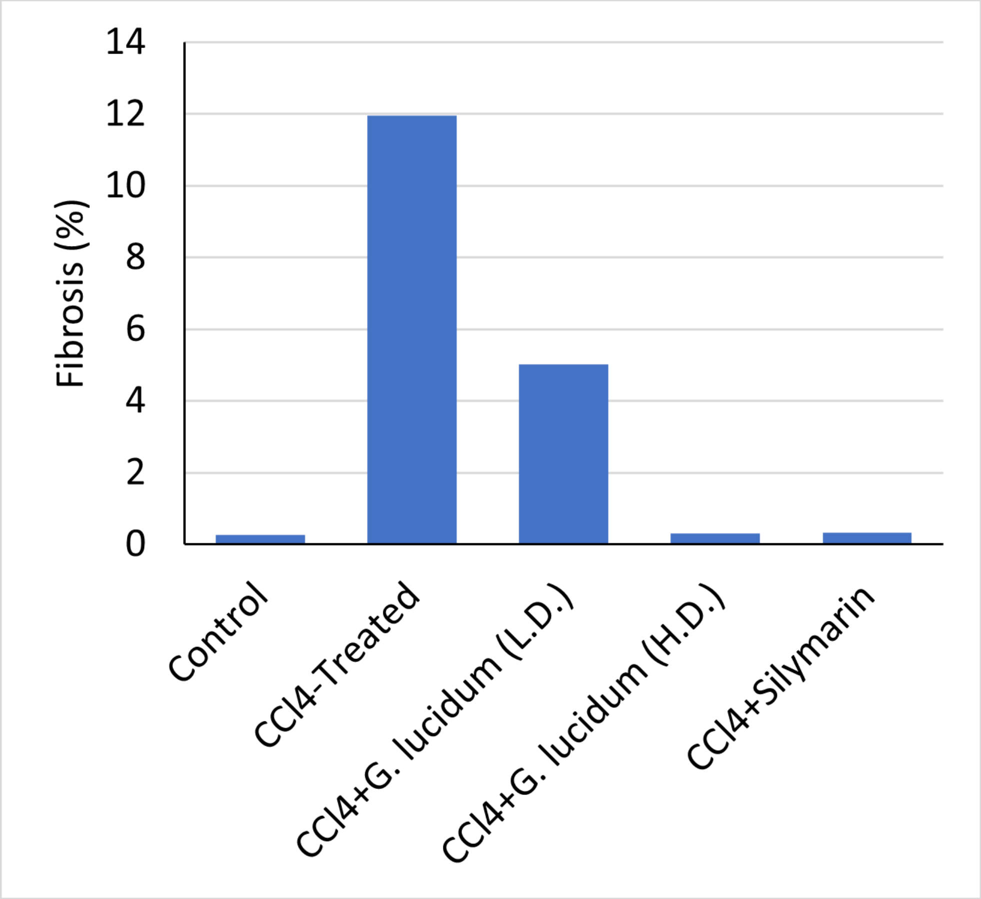

Fig. S1. Quantification of hepatic fibrosis

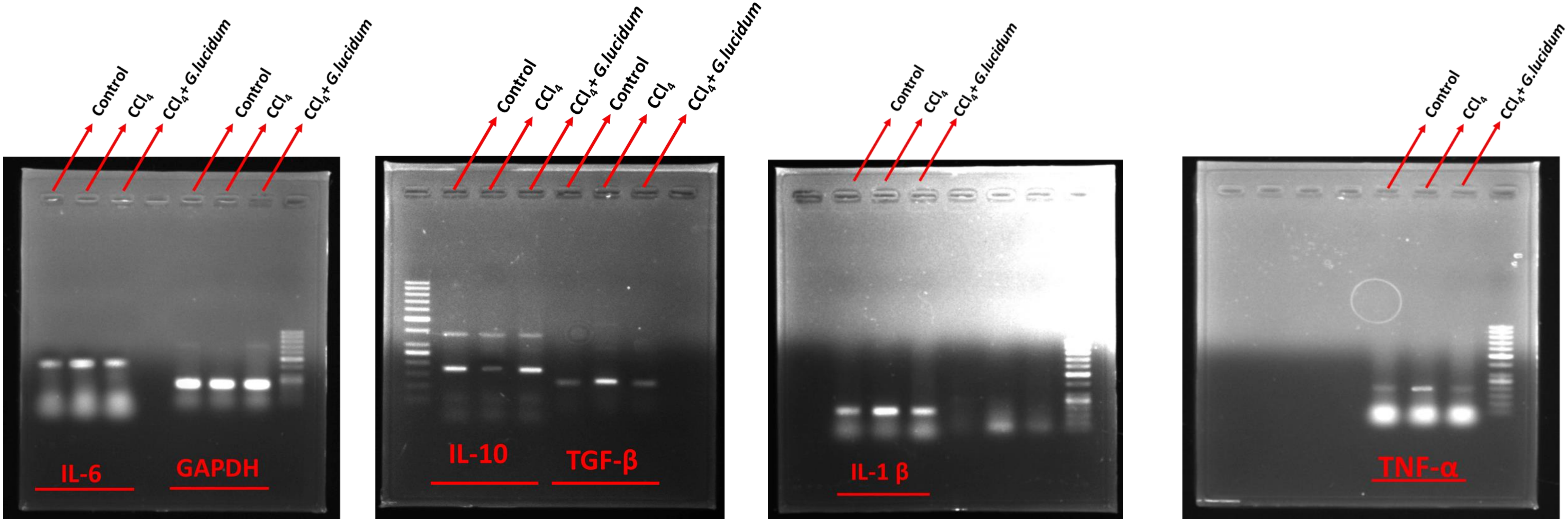

Fig. S2. Full length gels for gene expression analysis

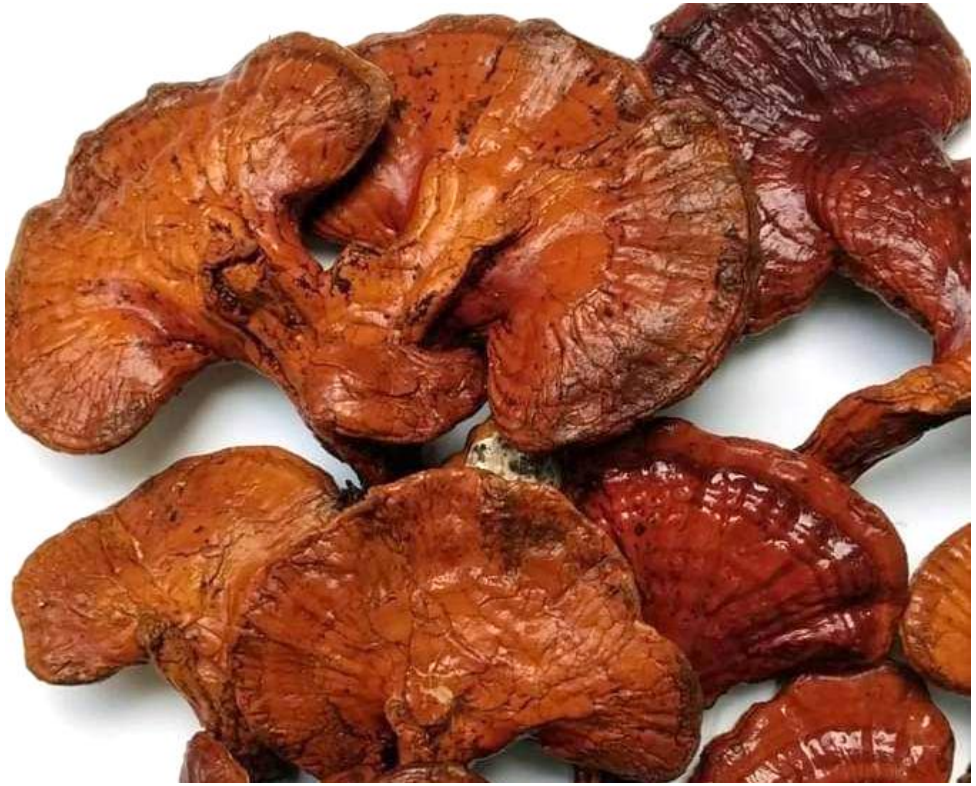

Fig. S3. Photograph of whole *G. lucidum*

Target Gene	Primer Sequence (5′–3′)	Annealing Temperature
**GAPDH**	F-5′-CTTGCCGTGGGTAGAGTCAT-3′<br>R-5′-AGACAGCCGCATCTTCTTGT-3′	F- 60.5 ^{o}C<br>R- 58.4 ^{o}C
**IL-1-β**	F-5′-AGACCTGACTTGGCAGAGA 3′<br>R-5′-GCAATGGTCGGGACATAGTT3′	F- 57.3 ^{o}C<br>R- 58.4 ^{o}C
**IL-6**	F-5′-CCCACCAGGAACGAAAGTCA3′<br>R-5′-GGCAACTGGCTGGAAGTCTC3′	F- 60.5 ^{o}C<br>R- 63.3 ^{o}C
**IL-10**	F-5′-ATCATGGAAGGAGCAACCTG3′<br>R-5′-GGGAAGCAACTGAAACTTCG3′	F- 58.4 ^{o}C<br>R- 58.4 ^{o}C
**TNF-α**	F-5′-TGTGGGTGAGGAGCACATAG-3′<br>R-5′-GTCGTAGCAAACCACCAAGC-3′	F- 60.5 ^{o}C<br>R- 60.5 ^{o}C
**TGF-β**	F-5′-AATTCCTGGCGTTACCT3′<br>R-5′-CCTGTATTCCGTCTCCTT3′	F- 50 ^{o}C<br>R- 53.9 ^{o}C

Table S1: The sequences of the primers used in the quantitative polymerase chain reaction (qPCR) assay
